# Supplementary material for: The Role of Gender Differences and Menopause in Obesity-Related Renal Disease, Renal Inflammation and Lipotoxicity
Source: Int J Mol Sci. 2023 Aug 19;24(16):12984. doi: 10.3390/ijms241612984 (PMC10455320; doi:10.3390/ijms241612984)
Supplement: Supplementary file 1 [file ijms-24-12984-s001.zip › ijms-2518934-SI.pdf]

THE ROLE OF GENDER DIFFERENCES AND MENOPAUSE IN OBESITY-RELATED RENAL DISEASE, RENAL INFLAMMATION AND LIPOTOXICITY

Supplementary material

Table S1: Dietary fatty acid profile.

Table S2: Lipid classes profile from kidney total lipids.

Table S3: Fatty acid profile from kidney total lipids.

Table S4: Fatty acid profile from kidney phosphatidylcholine.

Table S5: Fatty acid profile from kidney phosphatidylethanolamine.

Table S6: Fatty acid profile from kidney phosphatidylinositol.

Table S7: Fatty acid profile from kidney triglycerides.

Table S8: Lipid classes profile from urine total lipids.

Table S9: Fatty acid profile from urine total lipids.

Fatty acids	Standard Diet	Hight Fat Diet
**N**	**3**	**3**
**SFAs**	18.7 ± 0.3	**36.9 ± 0.3*** **
14:0	0.1 ± 0.0	**1.1 ± 0.0*** **
16:0	15.3 ± 0.3	**22.3 ± 0.1*** **
17:0	0.1 ± 0.0	**0.3 ± 0.0*** **
18:0	2.1 ± 0.0	**12.9 ± 0.1*** **
20:0	0.3 ± 0.1	0.2 ± 0.0
**MUFAs**	22.0 ± 0.2	**39.9 ± 0.1*** **
16:1 n-9	0.1 ± 0.0	**0.3 ± 0.0*** **
16:1 n-7	0.3 ± 0.0	**1.6 ± 0.0*** **
18:1 n-9	19.3 ± 0.1	**34.3 ± 0.2*** **
18:1 n-7	1.3 ± 0.1	**2.4 ± 0.1*** **
20:1 n-9	0.5 ± 0.0	**0.6 ± 0.0** **
**n-6 PUFAs**	54.2 ± 0.8	**20.2 ± 0.3*** **
18:2 n-6	54.1 ± 0.8	**19.4 ± 0.2*** **
20:2 n-6	0.1 ± 0.0	**0.5 ± 0.0*** **
20:4 n-6	nd	**0.2 ± 0.0** **
**n-3 PUFAs**	3.9 ± 0.2	**1.9 ± 0.3*** **
18:3 n-3	3.7 ± 0.1	**1.5 ± 0.0*** **
18:4 n-3	0.1 ± 0.0	0.1 ± 0.0
20:3 n-3	nd	**0.1 ± 0.0* **
20:5 n-3	0.1 ± 0.0	0.1 ± 0.0
22:5 n-3	nd	**0.1 ± 0.0** **
22:6 n-3	0.1 ± 0.0	**0.1 ± 0.0*** **
**n-3 LC-PUFAs**	0.2 ± 0.1	0.4 ± 0.3
**n-6 LC-PUFA**	nd	**0.3 ± 0.0*** **
**n-3 / n-6**	0.1 ± 0.0	**0.1 ± 0.0* **
**18:1 n-9 / 18:0**	9.3 ± 0.2	**2.6 ± 0.0*** **
**MUFAs / SFAs**	3.1 ± 0.0	**0.6 ± 0.0*** **
**PUFAs / MUFAs**	2.6 ± 0.1	**0.6 ± 0.0*** **
**PUFAs / SFAs**	3.1 ± 0.0	**0.6 ± 0.0*** **
**Total FA (g fatty acid / 100 g)**	2.8 ± 0.5	**20.3 ± 1.3*** **
**Total lipid (g lipid / 100 g)**	4.9 ± 0.2	**29.6 ± 2.0*** **

Table S1. Dietary fatty acid profile.

Results are given as mean ± standard deviation. Nd, not detected. SFAs, total saturated fatty acids; MUFAs, total monounsaturated fatty acids; n-6 and n-3 PUFAs, total omega-6 and total omega-3 polyunsaturated fatty acids, respectively; n-3 and n-6 LC-PUFA, total omega-3 and total omega-6 long-chain polyunsaturated fatty acids, respectively. * ($p < 0.05$); ** ($p < 0.01$); *** ($p < 0.001$).

Lipid classes	MALES		FEMALES			
	**SD**	**HFD**	**SD**	**SD-OVX**	**HFD**	**HFD-OVX**
**N**	**17**	**26**	**6**	**9**	**7**	**14**
Sphingomyelin	2.2 ± 0.5	2.4 ± 0.6	2.7 ± 1.4	2.2 ± 1.4	1.8 ± 1.3	2.1 ± 1.8
Phosphatidylcholine	15.9 ± 1.6	18.1 ± 2.7	15.6 ± 1.6	14.9 ± 3.5	15.6 ± 3.3	15.2 ± 3.7
Phosphatidylserine	3.0 ± 0.6	3.0 ± 0.5	4.4 ± 0.7	3.5 ± 0.8	3.8 ± 0.3	4.3 ± 1.4
Phosphatidylinositol	6.2 ± 0.8	6.3 ± 1.0	5.8 ± 1.1	5.2 ± 1.2	5.7 ± 0.3	5.2 ± 1.7
Phosphatidylglycerol	7.6 ± 1.2	6.3 ± 1.0	8.2 ± 1.3	6.5 ± 1.5	6.9 ± 0.4	6.7 ± 1.3
Phosphatidylethanolamine	16.3 ± 2.7	15.1 ± 2.7	16.9 ± 2.7	15.3 ± 3.2	16.4 ± 0.9	15.4 ± 1.4
**Total polar lipids**	54.3 ± 7.7	53.1 ± 7.8	57.8 ± 9.1	51.9 ± 12.0	54.6 ± 7.3	53.0 ± 12.5
Monoglycerides + Pigments	2.0 ± 0.4	1.8 ± 0.6	2.3 ± 0.9	1.8 ± 1.5	0.7 ± 1.1	1.5 ± 0.9
Cholesterol	15.4 ± 2.2	14.2 ± 1.9	17.5 ± 3.6	21.5 ± 4.7	23.28 ± 3.1	21.7 ± 6.8
Free fatty acids	4.4 ± 1.2	3.3 ± 1.6	5.7 ± 3.0	5.6 ± 3.0	3.4 ± 2.9	4.2 ± 0.7
Triglycerides	19.2 ± 7.5	16.7 ±10.6	12.7 ± 5.1	16.2 ± 8.1	15.2 ± 3.1	15.2 ± 6.4
Waxes + cholesterol esters	2.6 ± 1.1	3.9 ± 2.1	2.2 ± 1.2	1.2 ± 1.8	1.5 ± 2.5	2.27 ± 2.2
**Total neutral lipids**	43.7 ± 12.5	42.1 ± 8.9	37.0 ± 14.6	46.3 ±19.2	44.1 ± 12.6	44.9 ± 17.0
**Total lipid (g lipid /100 g renal tissue)**	5.6 ± 1.4	**6.9 ± 2.2***	5.0 ± 1.0	5.6 ± 3.3	4.9 ± 0.5	5.5 ± 2.3

Table S2. Lipid classes profile from kidney total lipids.

Results are given as mean ± standard deviation. SD, standard diet; HFD, high fat diet; OVX, ovariectomized. *, significant differences between males ($p < 0.05$).

Fatty acids	MALES		FEMALES			
	SD	HFD	SD	SD-OVX	HFD	HFD-OVX
**N**	**17**	**26**	**6**	**9**	**7**	**10**
**SFAs**	33.9 ± 1.2	29.0 ± 0.5	32.9 ± 0.9	30.0 ± 2.1	30.6 ± 1.4	30.6 ± 1.9
14:0	0.5 ± 0.1	0.5 ± 0.1	0.6 ± 0.1	0.4 ± 0.1	0.2 ± 0.1	0.3 ± 0.1
16:0	22.7 ± 1.7	**17.0 ± 0.4 *** **	19.3 ± 0.5	16.8 ± 2.2	16.7 ± 1.8	15.9 ± 1.3
18:0	10.4 ± 1.0	11.1 ± 0.4	12.6 ± 0.6	12.6 ± 1.4	13.4 ± 1.3	14.1 ± 1.0
20:0	0.3 ± 0.3	0.3 ± 0.1	0.4 ± 0.0	0.3 ± 0.1	0.3 ± 0.0	0.3 ± 0.0
**MUFAs**	20.4 ± 1.9	18.9 ± 1.4	23.7 ± 1.8	21.2 ± 3.2	18.3 ± 2.6	16.6 ± 1.4
16:1 n-7	2.2 ± 0.5	1.7 ± 0.3	2.4 ± 0.6	1.8 ± 0.4	**0.8 ± 0.1 a**	**0.9 ± 0.3 b**
18:1 n-9	17.6 ± 1.8	16.6 ± 1.1	20.8 ± 1.6	18.9 ± 3.0	17.2 ± 2.7	15.3 ± 3.7
20:1 n-9	0.6 ± 0.1	0.5 ± 0.1	0.5 ± 0.0	0.5 ± 0.1	0.4 ± 0.1	0.3 ± 0.1
**n-6 PUFAs**	31.1 ± 0.8	29.1 ± 0.5	33.3 ± 0.9	38.1 ± 3.0	36.0 ± 2.3	37.7 ± 1.3
18:2 n-6	15.9 ± 1.0	**13.0 ± 0.8***	14.5 ± 1.0	14.33 ± 1.8	12.36 ± 1.0	13.27 ± 1.6
20:2 n-6	0.3 ± 0.0	**0.7 ± 0.0*** **	0.4 ± 0.0	0.3 ± 0.1	**0.7 ± 0.1 a**	**0.6 ± 0.1 b**
20:3 n-6	1.1 ± 0.1	**0.7 ± 0.1*** **	0.7 ± 0.3	0.8 ± 0.1	0.6 ± 0.1	0.9 ± 0.2
20:4 n-6	13.8 ± 1.4	14.7 ± 0.7	17.7 ± 1.2	22.6 ± 3.1	22.3 ± 1.9	23.2 ± 1.5
**n-3 PUFAs**	14.6 ± 1.3	**23.0 ± 1.7*** **	10.1 ± 1.1	10.7 ± 1.3	**15.0 ± 1.8 a**	**15.0 ± 1.0 b**
18:3 n-3	0.4 ± 0.1	0.3 ± 0.3	0.2 ± 0.1	0.2 ± 0.1	0.2 ± 0.0	0.2 ± 0.0
22:6 n-3	14.2 ± 4.9	**22.7 ± 6.2*** **	9.8 ± 1.1	10.5 ± 1.4	**14.9 ± 1.8 a**	**14.8 ± 1.0 b**
**n-3 LC-PUFA**	14.2 ± 1.3	**23.0 ± 1.5*** **	9.9 ± 1.1	10.6 ± 1.5	**14.9 ± 1.8 a**	**14.8 ± 1.0 b**
**n-6 LC-PUFA**	15.2 ± 1.5	16.1 ± 0.7	**18.8 ± 1.3 c**	23.8 ± 3.2	23.6 ± 2.0	24.5 ± 1.4
**n-6/n-3**	2.4 ± 0.3	**1.5 ± 0.1*** **	3.5 ± 0.4	4.0 ± 0.6	2.6 ± 0.4	2.6 ± 0.2
**ARA/DHA**	0.9 ± 0.1	**0.7 ± 0.0*** **	1.8 ± 0.1	2.2 ± 0.1	**1.6 ± 0.2 d**	**1.6 ± 0.1 e**
**18:1 n-9 / 18:0**	2.4 ± 0.7	1.6 ± 0.2	1.7 ± 0.2	1.8 ± 0.5	1.4 ± 0.3	1.2 ± 0.2
**MUFAs / SFAs**	0.6 ± 0.1	0.6 ± 0.5	0.7 ± 0.1	0.7 ± 0.1	0.6 ± 0.1	0.6 ± 0.1
**PUFAs / MUFAs**	2.7 ± 0.4	3.2 ± 0.3	1.9 ± 0.2	3.0 ± 0.6	3.3 ± 0.7	3.3 ± 0.7
**PUFAs / SFAs**	1.4 ± 0.1	**1.8 ± 0.1*** **	1.3 ± 0.1	1.7 ± 0.2	1.7 ± 0.2	1.8 ± 0.1

Table S3. Fatty acids profile (% of total fatty acids) from kidney total lipids.

Results are given as mean ± standard deviation. SD, standard diet; HFD, high fat diet; OVX, ovariectomized. ARA, arachidonic acid (20:4 n-6); DHA, docosahexaneoic acid (22:6 n-3). Minor fatty acids (<0.1%) were excluded for the analysis. Male significance: * ($p < 0.05$); ** ($p < 0.01$); *** ($p < 0.001$). Female significance: **a** = HFD vs SD: $p < 0.05$ and vs SD-OVX: $p < 0.05$; **b** = HFD-OVX vs SD: $p < 0.05$ and vs SD-OVX: $p < 0.05$; **c** = SD vs HFD: $p < 0.05$ and vs HFD-OVX: $p < 0.01$; **d** = HFD vs SD-OVX: $p < 0.05$; **e** = HFD-OVX vs SD-OVX: $p < 0.05$.

Fatty acids	MALES		FEMALES			
	**SD**	**HFD**	**SD**	**SD-OVX**	**HFD**	**HFD-OVX**
**N**	**6**	**6**	**6**	**6**	**6**	**6**
**SFAs**	43.2 ± 2.9	39.8 ± 5.9	47.0 ± 2.1	**43.3 ± 1.2 a**	46.1 ± 2.1	46.2 ± 2.0
14:0	0.2 ± 0.0	0.2 ± 0.1	0.3 ± 0.1	0.2 ± 0.1	0.2 ± 0.0	0.2 ± 0.1
15:0	0.3 ± 0.2	0.3 ± 0.2	0.2 ± 0.2	0.2 ± 0.2	0.1 ± 0.1	0.1 ± 0.1
16:0	32.8 ± 2.0	29.6 ± 4.6	32.1 ± 2.1	29.5 ± 1.3	31.8 ± 1.	30.2 ± 1.3
17:0	0.2 ± 0.0	0.2 ± 0.0	0.3 ± 0.0	0.3 ± 0.0	0.3 ± 0.0	0.3 ± 0.0
18:0	9.3 ± 1.0	9.3 ± 2.0	13.8 ± 1.3	12.9 ± 0.6	13.5 ± 0.6	**15.2 ± 0.9 b**
20:0	0.1 ± 0.1	0.1 ± 0.1	0.2 ± 0.1	0.1 ± 0.1	0.2 ± 0.0	0.2 ± 0.0
**MUFAs**	10.9 ± 0.5	9.7 ± 1.6	12.4 ± 0.9	13.2 ± 1.2	11.4 ± 0.4	13.1 ± 1.1
16:1 n-9	0.3 ± 0.0	0.3 ± 0.1	0.3 ± 0.1	0.4 ± 0.1	0.3 ± 0.1	0.4 ± 0.1
16:1 n-7	0.6 ± 0.1	**0.4 ± 0.1****	0.6 ± 0.2	0.6 ± 0.1	**0.45 ± 0.1 c**	**0.3 ± 0.1 d**
18:1 n-9	6.5 ± 0.5	6.7 ± 1.4	8.4 ± 0.7	8.9 ± 0.5	8.2 ± 0.2	9.4 ± 0.9
18:1 n-7	2.9 ± 0.2	**1.9 ± 0.2*****	2.6 ± 0.1	2.7 ± 0.2	**2.1 ± 0.1 e**	**2.4 ± 0.1 f**
20:1 n-9	0.3 ± 0.1	**0.2 ± 0.0***	0.2 ± 0.1	0.3 ± 0.1	0.2 ± 0.0	0.3 ± 0.1
**n-6 PUFAs**	22.0 ± 1.3	**18.4 ± 1.3****	25.7 ± 1.9	26.5 ± 1.3	24.2 ± 1.4	24.6 ± 2.0
18:2 n-6	8.4 ± 0.8	**5.6 ± 0.7*****	8.6 ± 0.4	8.7 ± 0.4	**7.0 ± 0.2 g**	8.0 ± 0.7
18:3 n-6	0.1 ± 0.1	0.1 ± 0.1	0.2 ± 0.1	0.1 ± 0.1	0.1 ± 0.1	0.1 ± 0.0
20:2 n-6	0.3 ± 0.1	0.3 ± 0.0	0.4 ± 0.1	0.4 ± 0.1	0.4 ± 0.0	0.4 ± 0.0
20:3 n-6	1.2 ± 0.1	**0.6 ± 0.1*****	0.8 ± 0.1	0.9 ± 0.0	**0.6 ± 0.0 c**	**0.6 ± 0.0 d**
20:4 n-6	10.8 ± 1.4	11.1 ± 1.5	14.9 ± 1.7	15.8 ± 1.3	15.6 ± 1.2	14.9 ± 1.6
22:4 n-6	0.3 ± 0.0	0.3 ± 0.0	0.3 ± 0.0	0.4 ± 0.1	0.3 ± 0.0	0.3 ± 0.1
22:5 n-6	0.9 ± 0.3	**0.4 ± 0.2***	0.4 ± 0.1	0.3 ± 0.1	0.1 ± 0.1	0.2 ± 0.2
**n-3 PUFAs**	21.8 ± 3.8	**29.6 ± 6.7***	11.6 ± 1.8	13.5 ± 1.7	**15.5 ± 1.0 h**	13.3 ± 1.7
20:5 n-3	0.2 ± 0.0	0.1 ± 0.1	0.2 ± 0.1	0.2 ± 0.1	0.2 ± 0.1	0.1 ± 0.1
22:5 n-3	1.0 ± 0.3	1.1 ± 0.4	0.4 ± 0.4	0.4 ± 0.0	**0.5 ± 0.0 i**	**0.5 ± 0.0 j**
22:6 n-3	20.6 ± 3.6	**28.3 ± 6.3***	10.7 ± 2.4	12.7 ± 1.8	**14.7 ± 1.0 k**	12.7 ± 1.6
**DMAs**	0.6 ± 0.2	0.8 ± 0.3	1.0 ± 0.4	1.2 ± 0.3	1.3 ± 0.2	1.1 ± 0.4
16:0 DMA	0.3 ± 0.1	0.3 ± 0.1	0.6 ± 0.3	0.6 ± 0.3	0.5 ± 0.2	0.4 ± 0.1
18: 0 DMA	0.1 ± 0.1	0.1 ± 0.1	0.1 ± 0.1	0.1 ± 0.1	0.2 ± 0.1	0.2 ± 0.1
18:1 n-9 DMA	0.3 ± 0.1	0.4 ± 0.1	0.3 ± 0.2	0.5 ± 0.1	0.5 ± 0.1	0.5 ± 0.1
**n-3 LC-PUFA**	21.8 ± 3.8	29.6 ± 6.8	11.6 ± 1.8	13.5 ± 1.8	**15.5 ± 1.0 k**	13.3 ± 1.7
**n-6 LC-PUFA**	13.2 ± 1.2	12.5 ± 1.4	16.5 ± 1.7	17.3 ± 1.4	16.6 ± 1.3	16.1 ± 1.5
**n-6/n-3**	1.0 ± 0.2	**0.6 ± 0.1****	2.2 ± 0.2	2.0 ± 0.2	**1.6 ± 0.1 h**	**1.9 ± 0.1 l**
**ARA/DHA**	0.5 ± 0.1	0.4 ± 0.1	**1.4 ± 0.2 m**	1.2 ± 0.1	1.1 ± 0.1	1.2 ± 0.1
**18:1 n-9 / 18: 0**	0.7 ± 0.1	0.7 ± 0.2	0.6 ± 0.0	0.7 ± 0.1	0.6 ± 0.0	0.6 ± 0.0
**MUFAs / SFAs**	0.2 ± 0.0	0.2 ± 0.0	0.3 ± 0.0	**0.3 ± 0.0 n**	0.2 ± 0.0	0.3 ± 0.0
**PUFAs / MUFAs**	4.0 ± 0.5	5.1 ± 1.5	3.0 ± 0.5	3.1 ± 0.5	3.5 ± 0.3	2.9 ± 0.5
**PUFAs / SFAs**	1.0 ± 0.2	1.3 ± 0.4	0.8 ± 0.1	0.9 ± 0.1	0.9 ± 0.1	0.8 ± 0.1

Table S4. Fatty acid profile (% of total fatty acids) from kidney phosphatidylcholine.

Results are given as mean ± standard deviation. SD, standard diet; HFD, high fat diet; OVX, ovariectomized. DMAs, dimethyl-acetal. ARA, arachidonic acid (20:4 n-6); DHA, docosahexaenoic acid (22:6 n-3). Minor fatty acids (<0.1%) were excluded for the analysis. Male significance: * (p < 0.05); ** (p < 0.01); *** (p < 0.001). Female significance: **a** = SD-OVX vs SD: p < 0.05; **b** = HFD-OVX vs SD-OVX: p < 0.01; and vs HFD: p < 0.05; **c** = HFD vs SD: p < 0.001 and vs SD-OVX: p < 0.001 and vs HFD-OVX: p < 0.05; **d** = HFD-OVX vs SD: p < 0.05 and vs SD-OVX: p < 0.001 and vs HFD: p < 0.05. **e** = HFD vs SD: p < 0.05 and vs SD-OVX: p < 0.001 and vs HFD-OVX: p < 0.05; **f** = HFD-OVX vs SD-OVX: p < 0.05 and vs HFD: p < 0.001; **g** = HFD vs SD: p < 0.01 and vs SD-OVX: p < 0.001 and vs HFD-OVX: p < 0.05; **h** = HFD vs SD: p < 0.001 and vs SD-OVX: p < 0.01; **i** = HFD vs SD: p < 0.01 and vs SD-OVX: p < 0.01; **j** = HFD-OVX vs SD: p < 0.05 and vs SD-OVX: p < 0.05; **k** = HFD vs SD: p < 0.05; **l** = HFD-OVX vs SD: p < 0.01; **m** = SD vs HFD: p < 0.01 and vs HFD-OVX: p < 0.05; **n =** SD-OVX vs SD: p < 0.05 and vs HFD: p < 0.01.

**Fatty acids**	**MALES**		**FEMALES**			
	**SD**	**HFD**	**SD**	**SD-OVX**	**HFD**	**HFD-OVX**
**N**	**6**	**6**	**6**	**6**	**6**	**6**
SFAs	31.0 ± 1.8	31.0 ± 3.9	31.0 ± 3.6	29.9 ± 2.2	28.4 ± 1.3	27.3 ± 0.4
15:0	0.3 ± 0.3	0.2 ± 0.3	0.1 ± 0.2	0.2 ± 0.4	0.1 ± 0.1	0.1 ± 0.1
16:0	8.2 ± 0.6	8.4 ± 1.0	8.0 ± 0.7	8.0 ± 0.6	7.3 ± 1.0	7.0 ± 0.3
17:0	0.3 ± 0.0	0.3 ± 0.0	0.3 ± 0.0	0.2 ± 0.0	0.2 ± 0.0	0.2 ± 0.0
18:0	21.9 ± 0.9	21.8 ± 2.5	22.1 ± 3.0	21.1 ± 1.6	20.6 ± 0.5	19.7 ± 0.1
20:0	0.1 ± 0.1	0.1 ± 0.1	0.2 ± 0.1	0.2 ± 0.0	**0.1 ± 0.0 a**	0.1 ± 0.0
MUFAs	11.0 ± 0.8	10.3 ± 1.1	11.2 ± 1.1	11.2 ± 0.6	**9.6 ± 0.4 b**	10.6 ± 1.1
16:1 n-7	0.5 ± 0.1	0.4 ± 0.2	0.5 ± 0.2	0.4 ± 0.1	0.3 ± 0.1	0.2 ± 0.0
18:1 n-9	7.9 ± 0.9	8.3 ± 1.0	8.9 ± 0.6	8.9 ± 0.7	8.0 ± 0.5	8.7 ± 0.8
18:1 n-7	1.7 ± 0.3	**1.2 ± 0.2***	1.1 ± 0.1	1.4 ± 0.5	0.9 ± 0.0	1.1 ± 0.2
20:1 n-9	0.3 ± 0.0	**0.2 ± 0.0****	0.2 ± 0.0	0.2 ± 0.0	**0.1 ± 0.0 c**	0.2 ± 0.1
**n-6 PUFAs**	34.1 ± 1.7	32.3 ± 1.2	36.9 ± 0.9	36.0 ± 1.1	35.2 ± 2.0	35.6 ± 0.6
18:2 n-6	3.7 ± 0.4	**2.9 ± 0.4****	3.5 ± 0.3	3.4 ± 0.4	2.8 ± 0.2	3.1 ± 0.5
20:2 n-6	0.2 ± 0.2	0.2 ± 0.1	0.2 ± 0.3	0.1 ± 0.1	0.1 ± 0.1	0.1 ± 0.1
20:3 n-6	0.6 ± 0.1	**0.3 ± 0.0*****	0.4 ± 0.1	0.4 ± 0.1	**0.25 ± 0.0 b**	**0.3 ± 0.0 d**
20:4 n-6	27.8 ± 2.0	27.7 ± 1.3	31.0 ± 1.2	30.7 ± 1.0	30.8 ± 2.0	30.5 ± 0.3
22:4 n-6	0.8 ± 0.1	0.7 ± 0.1	0.9 ± 0.1	0.9 ± 0.1	**0.7 ± 0.0 e**	**0.7 ± 0.0 f**
22:5 n-6	1.0 ± 0.2	**0.5 ± 0.1*****	0.9 ± 0.3	0.6 ± 0.1	**0.5 ± 0.1 a**	0.6 ± 0.0
**n-3 PUFAs**	13.1 ± 1.1	15.2 ± 1.9	10.5 ± 1.8	10.4 ± 2.4	13.6 ± 0.4	13.0 ± 0.5
18:4 n-3	nd	nd	0.2 ± 0.2	0.1 ± 0.1	0.2 ± 0.1	0.4 ± 0.3
20:5 n-3	0.3 ± 0.0	0.3 ± 0.0	0.2 ± 0.1	0.2 ± 0.1	**0.3 ± 0.0 a**	0.3 ± 0.1
22:5 n-3	0.6 ± 0.1	0.5 ± 0.1	0.3 ± 0.2	0.3 ± 0.1	0.3 ± 0.0	0.4 ± 0.0
22:6 n-3	12.2 ± 1.1	**14.4 ± 1.8***	9.8 ± 1.9	9.7 ± 2.2	**12.6 ± 0.4 b**	11.9 ± 0.9
**DMAs**	8.2 ± 1.4	8.9 ± 2.5	7.9 ± 3.6	10.1 ± 1.2	11.4 ± 1.2	12.2 ± 1.2
16:0 DMA	3.2 ± 0.5	3.0 ± 0.9	2.9 ± 1.1	3.6 ± 0.4	3.7 ± 0.5	3.5 ± 0.4
18: 0 DMA	2.4 ± 0.5	3.2 ± 0.8	2.8 ± 1.2	3.4 ± 0.4	**4.9 ± 0.4 g**	**5.4 ± 0.7 f**
18:1 n-9 DMA	2.0 ± 0.4	2.4 ± 0.7	1.9 ± 0.9	2.5 ± 0.4	2.6 ± 0.3	2.8 ± 0.4
18:1 n-7 DMA	0.6 ± 0.0	0.4 ± 0.1	0.4 ± 0.2	**0.6 ± 0.1 h**	0.4 ± 0.0	0.4 ± 0.0
**n-3 LC-PUFA**	13.1 ± 1.1	15.2 ± 1.9	10.3 ± 2.1	10.3 ± 2.3	13.4 ± 0.4	12.6 ± 0.8
**n-6 LC-PUFA**	30.1 ± 1.9	**29.2 ± 1.4***	33.1 ± 1.1	32.6 ± 1.0	32.3 ± 1.9	32.1 ± 0.9
**n-6/n-3**	2.6 ± 0.2	**2.1 ± 0.3***	3.6 ± 0.7	3.6 ± 0.9	2.6 ± 0.1	2.7 ± 0.1
**ARA/DHA**	2.3 ± 0.1	**1.9 ± 0.3***	3.2 ± 0.7	3.3 ± 0.8	2.4 ± 0.1	2.6 ± 0.2
**18:1 n-9 / 18:0**	0.4 ± 0.0	0.4 ± 0.0	0.4 ± 0.0	0.4 ± 0.0	0.4 ± 0.0	0.4 ± 0.1
**MUFAs / SFAs**	0.3 ± 0.0	0.3 ± 0.0	0.4 ± 0.0	0.4 ± 0.0	0.3 ± 0.0	0.4 ± 0.0
**PUFAs / MUFAs**	4.3 ± 0.4	4.7 ± 0.7	4.3 ± 0.6	4.2 ± 0.4	**5.1 ± 0.4 a**	4.6 ± 0.5
**PUFAs / SFAs**	1.5 ± 0.1	1.6 ± 0.2	1.6 ± 0.2	1.6 ± 0.2	1.7 ± 0.1	1.8 ± 0.0

Table S5. Fatty acid profile (% of total fatty acids) from kidney phosphatidylethanolamine.

Results are given as mean ± standard deviation. SD, standard diet; HFD, high fat diet; OVX, ovariectomized. ARA, arachidonic acid (20:4 n-6); DHA, docosahexaenoic acid (22:6 n-3). Minor fatty acids (<0.1 %) were excluded for the analysis. Nd, not detected. Male significance: * ($p < 0.05$); ** ($p < 0.01$); *** ($p < 0.001$). Females significance: **a** = HFD vs SD: $p < 0.05$; **b** = HFD vs SD: $p < 0.05$ and vs SD-OVX: $p < 0.05$; **c** = HFD vs SD: $p < 0.01$ and vs SD-OVX: $p < 0.05$ and vs HFD-OVX: $p < 0.05$; **d** = HFD-OVX vs SD: $p < 0.05$ and vs SD-OVX: $p < 0.05$; **e** = HFD vs SD: $p < 0.01$ and vs SD-OVX: $p < 0.05$; **f** = HFD-OVX vs SD: $p < 0.01$ and vs SD-OVX: $p < 0.05$; **g** = HFD vs SD: $p < 0.01$ and vs SD-OVX: $p < 0.01$; **h** = SD-OVX vs HFD: $p < 0.01$ and vs HFD-OVX: $p < 0.05$.

Fatty acids	MALES		FEMALES			
	**SD**	**HFD**	**SD**	**SD-OVX**	**HFD**	**HFD-OVX**
**N**	**6**	**6**	**6**	**6**	**6**	**6**
**SFAs**	46.1 ± 6.0	43.3 ± 1.4	47.1 ± 5.1	46.1 ± 1.2	45.4 ± 1.6	45.0 ± 2.7
14:0	0.25 ± 0.17	0.23 ± 0.19	0.4 ± 0.3	0.3 ± 0.2	0.1 ± 0.2	0.3 ± 0.3
16:0	7.67 ± 2.1	7.16 ± 1.0	7.6 ± 1.9	7.7 ± 2.0	5.9 ± 1.9	7.0 ± 1.9
17:0	0.3 ± 0.1	0.2 ± 0.1	0.2 ± 0.3	0.2 ± 0.1	0.2 ± 0.1	0.2 ± 0.1
18:0	36.7 ± 3.7	34.3 ± 2.4	38.2 ± 3.8	37.1 ± 1.6	38.6 ± 2.6	37.2 ± 3.3
20:0	0.3 ± 0.1	0.2 ± 0.1	0.2 ± 0.1	0.2 ± 0.1	0.2 ± 0.1	0.1 ± 0.1
**MUFAs**	6.7 ± 3.1	7.3 ± 2.1	7.9 ± 2.9	7.9 ± 1.8	5.8 ± 1.3	7.4 ± 3.8
16:1 n-9	0.2 ± 0.2	0.2 ± 0.2	nd	0.2 ± 0.2	nd	nd
16:1 n-7	0.4 ± 0.1	0.4 ± 0.2	0.6 ± 0.4	0.5 ± 0.1	0.4 ± 0.2	0.4 ± 0.4
18:1 n-9	4.3 ± 1.9	4.9 ± 1.7	5.9 ± 2.2	5.9 ± 1.5	4.1 ± 0.8	5.2 ± 2.6
18:1 n-7	1.3 ± 0.4	1.5 ± 0.6	1.0 ± 0.2	1.0 ± 0.1	0.9 ± 0.2	1.0 ± 0.4
20:1 n-9	0.1 ± 0.2	0.1 ± 0.1	nd	nd	0.2 ± 0.3	nd
**n-6 PUFAs**	33.8 ± 5.5	35.3 ± 2.7	33.9 ± 4.9	34.9 ± 3.3	37.4 ± 3.3	38.7 ± 3.8
18:2 n-6	4.3 ± 1.1	6.1 ± 2.2	3.4 ± 0.6	3.4 ± 0.7	2.7 ± 0.4	3.6 ± 1.0
18:3 n-6	0.2 ± 0.3	0.2 ± 0.2	0.3 ± 0.3	0.2 ± 0.2	0.3 ± 0.2	0.4 ± 0.4
20:2 n-6	0.2 ± 0.1	**0.5 ± 0.2***	0.2 ± 0.2	0.3 ± 0.2	0.2 ± 0.1	0.2 ± 0.1
20:3 n-6	3.4 ± 1.2	**2.0 ± 0.3***	1.7 ± 0.4	1.9 ± 0.1	**1.0 ± 0.1 a**	**1.0 ± 0.2 b**
20:4 n-6	23.8 ± 4.9	25.4 ± 3.0	27.1 ± 5.4	28.1 ± 3.4	32.3 ± 3.6	32.5 ± 4.7
22:4 n-6	0.6 ± 0.2	0.6 ± 0.1	0.6 ± 0.1	0.5 ± 0.3	0.6 ± 0.1	0.5 ± 0.1
22:5 n-6	0.8 ± 0.3	0.4 ± 0.1	0.6 ± 0.2	0.4 ± 0.2	0.4 ± 0.1	0.6 ± 0.2
**n-3 PUFAs**	9.0 ± 4.0	10.0 ± 0.8	5.5 ± 1.2	6.3 ± 0.7	7.1 ± 0.8	5.8 ± 0.5
18:3 n-3	0.1 ± 0.1	0.1 ± 0.1	nd	nd	nd	nd
18:4 n-3	0.2 ± 0.2	0.2 ± 0.1	0.5 ± 0.5	0.3 ± 0.3	0.5 ± 0.3	0.3 ± 0.2
20:3 n-3	0.2 ± 0.2	0.2 ± 0.1	0.1 ± 0.1	0.4 ± 0.3	0.1 ± 0.0	0.1 ± 0.0
20:4 n-3	0.1 ± 0.2	nd	0.2 ± 0.1	0.2 ± 0.1	0.1 ± 0.1	nd
20:5 n-3	0.1 ± 0.1	0.2 ± 0.2	0.1 ± 0.1	0.1 ± 0.1	0.4 ± 0.4	0.2 ± 0.1
22:5 n-3	0.5 ± 0.3	0.6 ± 0.2	0.2 ± 0.1	0.3 ± 0.2	0.3 ± 0.0	0.2 ± 0.2
22:6 n-3	7.8 ± 3.9	8.7 ± 0.8	4.5 ± 1.2	4.8 ± 0.8	5.6 ± 0.7	5.3 ± 0.4
**DMAs**	0.5 ± 0.4	0.5 ± 0.5	0.5 ± 0.4	0.4 ± 0.4	0.7 ± 0.2	0.3 ± 0.3
16:0 DMA	0.2 ± 0.2	0.2 ± 0.2	0.3 ± 0.2	0.2 ± 0.1	0.2 ± 0.1	0.2 ± 0.2
18: 0 DMA	0.2 ± 0.1	0.2 ± 0.1	0.1 ± 0.1	0.1 ± 0.1	0.3 ± 0.2	0.1 ± 0.1
18:1 n-9 DMA	0.2 ± 0.2	0.2 ± 0.1	0.2 ± 0.1	0.2 ± 0.1	0.2 ± 0.1	0.1 ± 0.0
**n-3 LC-PUFA**	8.8 ± 4.1	9.7 ± 0.8	4.9 ± 1.1	5.9 ± 0.9	6.6 ± 0.7	5.6 ± 0.6
**n-6 LC-PUFA**	29.1 ± 6.1	28.5 ± 3.3	30.1 ± 5.7	30.9 ± 3.8	34.2 ± 3.7	34.6 ± 4.8
**n-6/n-3**	4.2 ± 1.3	3.6 ± 0.4	6.4 ± 1.6	5.9 ± 0.5	5.3 ± 0.9	6.7 ± 0.5
**ARA/DHA**	3.5 ± 0.9	2.9 ± 0.5	6.2 ± 0.9	5.8 ± 0.3	5.8 ± 0.7	6.2 ± 0.7
**18:1 n-9 / 18:0**	0.1 ± 0.0	0.1 ± 0.1	0.2 ± 0.1	0.2 ± 0.1	0.1 ± 0.0	0.2 ± 0.1
**MUFAs / SFAs**	0.1 ± 0.1	0.2 ± 0.1	0.2 ± 0.1	0.2 ± 0.0	0.1 ± 0.0	0.2 ± 0.1
**PUFAs / MUFAs**	7.5 ± 3.5	6.8 ± 2.5	5.5 ± 1.9	5.6 ± 1.8	8.0 ± 2.2	7.6 ± 4.0
**PUFAs / SFAs**	1.0 ± 0.3	1.0 ± 0.1	0.8 ± 0.2	0.9 ± 0.1	1.0 ± 0.1	1.0 ± 0.1

Table S6. Fatty acid profile (% of total fatty acids) from kidney phosphatidylinositol.

Results are given as mean ± standard deviation. SD, standard diet; HFD, high fat diet; OVX, ovariectomized. ARA, arachidonic acid (20:4 n-6); DHA, docosahexaenoic acid (22:6 n-3). Minor fatty acids (<0.1%) were excluded for the analysis. Nd, not detected. Male significance: * ($p < 0.05$). Female significance: **a** = HFD vs SD: $p < 0.01$ and vs SD-OVX: $p < 0.001$; **b =** HFD-OVX vs SD: $p < 0.01$ and vs SD-OVX: $p < 0.001$.

Fatty acids	MALES		FEMALES			
	SD	HFD	SD	SD-OVX	HFD	HFD-OVX
N	6	6	6	6	6	6
**SFAs**	30.3 ± 2.4	**24.9 ± 1.6****	30.8 ± 3.8	29.3 ± 3.7	26.5 ± 1.0	27.4 ± 1.7
14:0	1.5 ± 0.3	**0.8 ± 0.1****	**1.4 ± 0.1 a**	1.0 ± 0.5	0.9 ± 0.0	0.9 ± 0.1
15:0	0.2 ± 0.1	**0.1 ± 0.0***	0.2 ± 0.0	0.2 ± 0.1	0.1 ± 0.0	**0.1 ± 0.0 b**
16:0	24.2 ± 2.0	**19.0 ± 0.8****	22.7 ± 2.4	21.1 ± 1.8	**18.7 ± 0.2 c**	**17.4 ± 0.6 d**
17:0	0.2 ± 0.0	**0.2 ± 0.0****	0.2 ± 0.0	0.2 ± 0.0	**0.3 ± 0.1 c**	**0.3 ± 0.0 e**
18:0	3.8 ± 1.3	4.5 ± 1.0	6.0 ± 1.3	5.9 ± 1.4	6.1 ± 0.8	**8.4 ± 1.4 f**
20:0	0.3 ± 0.1	**0.1 ± 0.0***	0.3 ± 0.2	0.3 ± 0.1	0.2 ± 0.1	0.3 ± 0.0
**MUFAs**	44.9 ± 3.0	**52.3 ± 2.3****	46.9 ± 2.9	47.0 ± 3.3	50.0 ± 3.0	50.6 ± 1.7
16:1 n-9	0.6 ± 0.1	**0.9 ± 0.1*****	0.7 ± 0.1	0.8 ± 0.1	0.7 ± 0.1	0.8 ± 0.1
16:1 n-7	6.9 ± 1.8	**3.5 ± 0.4****	5.1 ± 0.9	4.3 ± 0.9	**2.9 ± 0.4 g**	**1.9 ± 0.5 h**
18:1 n-9	31.5 ± 3.1	**43.8 ± 2.7*****	37.1 ± 2.8	36.9 ± 3.3	**42.1 ± 2.5 c**	**42.8 ± 2.1 f**
18:1 n-7	3.5 ± 0.5	2.8 ± 0.4	2.9 ± 0.6	3.5 ± 0.3	3.0 ± 0.1	3.2 ± 0.3
20:1 n-9	1.4 ± 0.8	**0.6 ± 0.1****	0.7 ± 0.1	0.9 ± 0.2	0.6 ± 0.0	**1.2 ± 0.3 i**
**n-6 PUFAs**	22.0 ± 3.7	20.3 ± 0.5	20.2 ± 1.8	20.4 ± 2.4	19.6 ± 0.5	19.7 ± 0.6
18:2 n-6	20.7 ± 3.6	18.8 ± 0.5	19.1 ± 1.6	19.3 ± 2.4	17.7 ± 0.4	17.9 ± 0.8
18:3 n-6	0.1 ± 0.1	0.1 ± 0.0	0.1 ± 0.1	nd	0.1 ± 0.2	0.1 ± 0.1
20:2 n-6	0.2 ± 0.0	**0.5 ± 0.0*****	0.2 ± 0.0	0.2 ± 0.0	**0.5 ± 0.0 j**	**0.6 ± 0.0 k**
20:3 n-6	0.2 ± 0.0	0.2 ± 0.0	0.1 ± 0.0	0.1 ± 0.1	0.2 ± 0.1	0.2 ± 0.0
20:4 n-6	0.5 ± 0.3	0.6 ± 0.1	0.4 ± 0.1	0.6 ± 0.2	0.8 ± 0.4	0.6 ± 0.2
22:4 n-6	0.1 ± 0.1	0.1 ± 0.1	0.1 ± 0.3	0.1 ± 0.1	0.2 ± 0.1	0.2 ± 0.1
22:5 n-6	0.1 ± 0.1	**0.1 ± 0.0***	0.1 ± 0.1	0.1 ± 0.1	0.1 ± 0.0	0.1 ± 0.1
**n-3 PUFAs**	1.5 ± 1.0	1.4 ± 0.4	1.0 ± 0.4	1.3 ± 0.7	2.0 ± 1.6	1.1 ± 0.3
18:3 n-3	0.7 ± 0.1	0.7 ± 0.1	0.5 ± 0.1	0.6 ± 0.2	**0.7 ± 0.1 l**	0.6 ± 0.1
18:4 n-3	0.1 ± 0.1	0.1 ± 0.1	0.1 ± 0.1	0.1 ± 0.1	0.1 ± 0.1	0.1 ± 0.0
20:5 n-3	0.2 ± 0.2	0.1 ± 0.1	0.1 ± 0.1	0.1 ± 0.1	0.5 ± 0.4	0.1 ± 0.0
22:5 n-3	0.1 ± 0.1	0.1 ± 0.1	nd	nd	0.2 ± 0.1	0.1 ± 0.1
22:6 n-3	0.6 ± 1.0	0.4 ± 0.3	0.3 ± 0.2	0.4 ± 0.3	0.6 ± 0.4	0.4 ± 0.1
**n-3 LC-PUFA**	0.8 ± 1.0	0.6 ± 0.4	0.4 ± 0.3	0.6 ± 0.5	1.2 ± 1.0	0.5 ± 0.3
**n-6 LC-PUFA**	1.0 ± 0.3	0.9 ± 0.2	0.8 ± 0.2	0.9 ± 0.2	1.3 ± 0.6	1.1 ± 0.3
**n-6/n-3**	17.8 ± 7.5	15.3 ± 3.6	22.0 ± 4.9	19.7 ± 9.9	13.3 ± 6.7	18.8 ± 4.3
**ARA/DHA**	1.6 ± 0.8	1.5 ± 0.6	2.2 ± 1.0	1.8 ± 0.8	1.4 ± 0.3	1.6 ± 0.2
**18:1 n-9 / 18:0**	8.9 ± 2.8	10.1 ± 2.6	6.5 ± 1.7	6.6 ± 1.8	7.0 ± 1.3	5.2 ± 1.0
**MUFAs / SFAs**	1.5 ± 0.1	2.1 ± 0.2	1.5 ± 0.3	1.6 ± 0.3	1.9 ± 0.2	1.9 ± 0.2
**PUFAs / MUFAs**	0.5 ± 0.1	0.4 ± 0.0	0.4 ± 0.0	0.5 ± 0.0	0.4 ± 0.1	0.4 ± 0.2
**PUFAs / SFAs**	0.8 ± 0.2	0.9 ± 0.1	0.7 ± 0.1	0.8 ± 0.2	0.8 ± 0.1	0.8 ± 0.1

Table S7. Fatty acid profile (% of total fatty acids) from kidney triglycerides.

Results are given as mean ± standard deviation. SD, standard diet; HFD, high fat diet; OVX, ovariectomized. ARA, arachidonic acid (20:4 n-6); DHA, docosahexaenoic acid (22:6 n-3). Minor fatty acids (<0.1%) were excluded for the analysis. Nd, not detected. Male significance: * ($p < 0.05$); ** ($p < 0.01$); *** ($p < 0.001$). Female significance: **a** = SD vs SD-OVX: $p < 0.05$ and vs HFD: $p < 0.05$ and vs HFD-OVX: $p < 0.01$; **b** = HFD-OVX vs SD-OVX: $p < 0.05$; **c** = HFD vs SD: $p < 0.05$ and vs SD-OVX: $p < 0.05$; **d** = HFD-OVX vs SD: $p < 0.05$ and vs SD-OVX: $p < 0.05$ and vs HFD: $p < 0.05$; **e** = HFD-OVX vs SD: $p < 0.01$ and vs SD-OVX: $p < 0.01$; **f** = HFD-OVX vs SD: $p < 0.05$ and vs SD-OVX: $p < 0.05$; **g** = HFD vs SD: $p < 0.01$ and vs SD-OVX: $p < 0.05$; **h** = HFD-OVX vs SD: $p < 0.001$ and vs SD-OVX: $p < 0.001$; **i** = HFD-OVX vs SD: $p < 0.05$ and vs HFD: $p < 0.01$; **j** = HFD vs SD: $p < 0.001$ and vs SD-OVX: $p < 0.001$; **k** = HFD-OVX vs SD: $p < 0.05$ and vs SD-OVX: $p < 0.05$ and vs HFD: $p < 0.05$; **l** = HFD vs SD: $p < 0.05$ and vs HFD-OVX: $p < 0.05$.

Lipid classes	MALES		FEMALES			
	**SD**	**HFD**	**SD**	**SD-OVX**	**HFD**	**HFD-OVX**
**N**	**7**	**6**	**6**	**9**	**8**	**8**
Sphingomyelin	1.3 ± 1.0	2.5 ± 1.4	2.3 ± 3.1	0.7 ± 0.3	2.4 ± 1.2	**2.3 ± 1.1 a**
Phosphatidylcholine	9.2 ± 2.7	**16.5 ± 5.1****	7.2 ± 1.0	**11.9 ± 3.6 b**	**12.1 ± 6.1 c**	**14.5 ± 4.6 a**
Phosphatidylserine	3.9 ± 2.7	6.8 ± 3.9	3.1 ± 3.3	2.4 ± 1.2	5.1 ± 4.6	5.3 ± 3.7
Phosphatidylinositol	2.8 ± 1.3	2.9 ± 1.3	1.9 ± 1.3	2.2 ± 1.3	2.8 ±1.63	**3.1 ± 1.1 a**
Phosphatidylglycerol	2.6 ± 1.4	3.7 ± 2.0	2.4 ± 1.3	3.2 ± 1.5	2.4 ± 1.2	3.1 ± 1.5
Phosphatidylethanolamine	9.7 ± 3.4	**18.5 ± 3.3 ****	8.6 ± 3.8	7.9 ± 2.6	8.3 ± 2.9	9.5 ± 2.3
**Total polar lipids**	46.7 ± 7.0	**62.7 ± 6.1****	43.2 ± 12.9	45.4 ± 10.9	47.6 ± 8.7	49.9 ± 6.2
Monoglycerides + Pigments	20.8 ± 5.9	**11.7 ± 3.7****	20.8 ± 4.3	18.5 ± 2.0	**15.3 ± 2.8 c**	**15.8 ± 4.5 a**
Diglycerides	1.3 ± 0.7	1.0 ± 0.7	2.0 ± 2.3	1.3 ± 0.3	1.2 ± 0.5	2.9 ± 3.8
Cholesterol	5.8 ± 0.9	**8.2 ± 1.3 ***	6.8 ± 2.3	6.4 ± 2.1	9.4 ± 2.2	9,0 ± 2.4
Free fatty acids	12.4 ± 4.6	**5.8 ± 2.5***	8.7 ± 4.1	8.0 ± 2.9	8.1 ± 2.5	6.9 ± 3.1
Triglycerides	6.3 ± 3.4	6.2 ± 1.9	9.3 ± 6.8	10.8 ± 7.6	10.6 ± 3.5	8.9 ± 5.2
Waxes + cholesterol esters	6.7 ± 2.5	4.3 ± 2.0	9.1 ± 2.8	9.6 ± 4.3	7.9 ± 4.1	6.5 ± 3.5
**Total neutral lipids**	53.3 ± 7.0	**37.3 ± 6.1***	56.8 ± 12.9	54.6 ± 10.9	52.4 ± 8.7	50.1 ± 6.2
**Total lipid (g /100 mL urine)**	0.2 ± 0.1	0.1 ± 0.1	0.5 ± 0.2	0.5 ± 0.4	0.6 ± 0.7	0.6 ± 0.6

Table S8. Lipid classes profile from urine.

Results are given as mean ± standard deviation. SD, standard diet; HFD, high fat diet; OVX, ovariectomized. Male significance: * ($p < 0.05$); ** ($p < 0.01$). Female significance: **a** = HFD-OVX vs SD-OVX: $p < 0.01$; **b** = SD-OVX vs SD: $p < 0.001$; **c** = HFD vs SD: $p < 0.05$: **d** = HFD vs SD-OVX: $p < 0.01$.

Fatty acids	MALES		FEMALES			
	**SD**	**HFD**	**SD**	**SD-OVX**	**HFD**	**HFD-OVX**
**N**	**7**	**6**	**6**	**9**	**8**	**15**
**SFAs**	51.7 ± 17.9	33.4 ± 13.2	57.0 ± 15.4	48.9 ± 20.9	44.3 ± 4.3	44.9 ± 6.4
14:0	0.7 ± 0.2	0.8 ± 0.3	1.1 ± 0.4	1.0 ± 0.4	1.1 ± 0.4	1.3 ± 0.3
15:0	1.7 ± 0.9	1.0 ± 0.4	2.1 ± 1.1	1.6 ± 0.6	2.4 ± 2.1	2.1 ± 2.0
16:0	22.4 ± 4.4	**16.1 ± 5.9 ***	26.4 ± 2.4	24.4 ± 5.6	**22.0 ± 1.8 a**	23.9 ± 3.8
17:0	0.4 ± 0.5	nd	0.4 ± 0.1	0.4 ± 0.1	0.5 ± 0.1	0.3 ± 0.4
18:0	25.0 ± 14.4	13.9 ± 6.9	25.2 ± 14.4	20.1 ± 15.9	16.0 ± 3.3	15.9 ± 3.1
20:0	0.7 ± 0.1	**0.6 ± 0.1 ***	0.9 ± 0.1	**0.7 ± 0.2 b**	0.8 ± 0.2	0.7 ± 0.2
**MUFAs**	15.1 ± 5.1	15.7 ± 2.2	19.4 ± 8.6	21.5 ± 8.8	23.5 ± 5.0	23.2 ± 5.1
16:1 n-9	0.5 ± 0.2	0.4 ± 0.2	0.6 ± 0.2	0.4 ± 0.1	0.6 ± 0.4	0.6 ± 0.5
16:1 n-7	0.4 ± 0.1	0.4 ± 0.1	0.5 ± 0.2	0.5 ± 0.2	0.7 ± 0.2	0.8 ± 0.2
18:1 n-9	11.5 ± 4.4	11.6 ± 2.3	13.3 ± 5.8	14.4 ± 7.1	18.2 ± 3.9	18.9 ± 5.3
18:1 n-7	1.4 ± 0.4	1.4 ± 0.3	1.5 ± 0.6	1.7 ± 0.7	1.8 ± 0.8	1.8 ± 0.6
20:1 n-9	0.8 ± 0.3	0.7 ± 0.2	1.2 ± 1.0	1.1 ± 1.3	1.0 ± 1.0	0.5 ± 0.2
20:1 n-7	0.2 ± 0.0	0.2 ± 0.1	0.8 ± 1.2	1.3 ± 1.1	**0.2 ± 0.1 c**	**0.1 ± 0.0 d**
24: 1 n-9	0.3 ± 0.2	**0.7 ± 0.1 *****	0.6 ± 0.6	0.4 ± 0.4	0.5 ± 0.3	0.4 ± 0.2
**n-6 PUFAs**	12.9 ± 5.2	8.9 ± 1.7	10.6 ± 4.0	12.4 ± 6.1	11.2 ± 1.4	10.2 ± 2.4
18:2 n-6	9.6 ± 4.1	**4.7 ± 1.3 ***	7.0 ± 2.6	9.3 ± 5.2	6.9 ± 1.3	**5.9 ± 1.5 e**
18:3 n-6	0.5 ± 0.2	0.2 ± 0.2	0.5 ± 0.6	0.6 ± 0.3	0.3 ± 0.4	**0.3 ± 0.2 f**
20:2 n-6	0.1 ± 0.1	**0.2 ± 0.0 ***	0.2 ± 0.2	0.2 ± 0.1	0.3 ± 0.4	**0.3 ± 0.3 d**
20:4 n-6	1.6 ± 0.5	**3.3 ± 0.8 *****	1.9 ± 1.0	1.7 ± 1.4	3.3 ± 1.5	3.1 ± 2.0
22:4 n-6	0.1 ± 0.1	0.2 ± 0.1	0.5 ± 0.4	0.2 ± 0.3	0.2 ± 0.2	0.2 ± 0.2
**n-3 PUFAs**	13.1 ± 6.0	**33.5 ± 11.3 ****	7.3 ± 2.5	6.9 ± 4.0	11.5 ± 4.8	11.0 ± 4.8
18:3 n-3	1.0 ± 0.6	0.7 ± 0.3	1.0 ± 0.6	1.0 ± 0.6	1.1 ± 0.2	1.2 ± 0.7
18:4 n-3	0.5 ± 0.4	0.4 ± 0.1	0.6 ± 0.3	0.7 ± 0.4	0.7 ± 0.3	0.8 ± 0.3
20:5 n-3	0.2 ± 0.1	0.3 ± 0.1	0.7 ± 1.1	0.8 ± 0.9	0.4 ± 0.2	0.3 ± 0.3
22:5 n-3	0.4 ± 0.1	**0.7 ± 0.3 ****	0.4 ± 0.2	0.3 ± 0.2	0.4 ± 0.2	0.4 ± 0.2
22:6 n-3	11.0 ± 5.1	**31.3 ± 11.1 *****	4.2 ± 1.6	4.2 ± 3.1	8.9 ± 4.6	8.3 ± 5.0
**DMAs**	2.3 ± 0.9	**3.7 ± 0.9 ***	2.5 ± 1.3	2.6 ± 2.2	3.4 ± 1.6	3.3 ± 1.8
18:0 DMA	0.4 ± 0.1	**0.7 ± 0.2 ****	0.9 ± 1.1	1.1 ± 2.0	1.0 ± 0.5	0.9 ± 0.5
18:1 n-9 DMA	1.2 ± 0.4	**2.2 ± 0.5 ****	1.2 ± 0.6	1.2 ± 0.8	2.1 ± 1.0	2.0 ± 1.2
**n-3 LC-PUFA**	11.6 ± 5.3	**32.4 ± 11.3 *****	5.6 ± 2.0	5.2 ± 3.7	9.7 ± 4.8	9.0 ± 5.2
**n-6 LC-PUFA**	2.9 ± 0.9	**4.0 ± 0.7 ***	3.0 ± 1.6	2.4 ± 1.7	4.0 ± 1.7	3.9 ± 2.2
**n-6/n-3**	1.0 ± 0.4	**0.3 ± 0.1 *****	1.4 ± 0.3	**2.0 ± 0.7 b**	1.3 ± 1.0	**1.1 ± 0.4 g**
**ARA/EPA**	7.3 ± 1.7	11.0 ± 4.8	8.2 ± 6.6	4.0 ± 3.4	9.3 ± 4.4	**11.0 ± 7.4 h**
**ARA/DHA**	0.2 ± 0.0	**0.1 ± 0.0 ***	0.5 ± 0.2	0.5 ± 0.2	0.4 ± 0.1	0.4 ± 0.1
**DHA/EPA**	48.6 ± 12.7	106.5 ± 59.4	21.0 ± 14.3	10.9 ± 11.8	26.3 ± 13.6	**30.0 ± 22.3 i**
**18:1 n-9 / 18:0**	0.8 ± 0.6	1.0 ± 0.4	0.7 ± 0.5	1.2 ± 0.7	1.2 ± 0.4	1.3 ± 0.5
**Total FA (g /100 mL urine)**	12.0 ± 9.6	6.3 ± 1.8	35.2 ± 35.0	38.1 ± 42.2	**19.7 ± 30.5 c**	22.5 ± 30.0

Table S9. Fatty acids profile from urine.

Results are given as mean ± standard deviation. SD, standard diet; HFD, high fat diet; OVX, ovariectomized. ARA, arachidonic acid (20:4 n-6); EPA, eicosapentaenoic acid (20:5 n-3); DHA, docosahexaenoic acid (22:6 n-3). Minor fatty acids (<0.1 %) were excluded for the analysis. Nd, not detected. Male significance: * ($p < 0.05$); ** ($p < 0.01$); *** ($p < 0.001$). Female significance: **a** = HFD vs SD: $p < 0.01$; **b** = SD-OVX vs SD: $p < 0.05$; **c** = HFD vs. SD: $p < 0.05$; **d** = HFD-OVX vs. SD-OVX: $p < 0.05$; **e** = HFD-OVX vs SD-OVX: $p < 0.05$ and vs HFD: $p < 0.05$; **f** = HFD-OVX vs SD-OVX: $p < 0.01$; **g** = HFD-OVX vs SD-OVX: $p < 0.001$; **h** < HFD-OVX vs SD-OVX: $p < 0.01$; **i** = HFD-OVX vs SD-OVX: $p < 0.05$.
